# Modulation by Sigma-1 Receptor of Morphine Analgesia and Tolerance: Nociceptive Pain, Tactile Allodynia and Grip Strength Deficits During Joint Inflammation

**DOI:** 10.3389/fphar.2019.00136

**Published:** 2019-02-22

**Authors:** Ángeles Montilla-García, Miguel Á. Tejada, M. Carmen Ruiz-Cantero, Inmaculada Bravo-Caparrós, Sandra Yeste, Daniel Zamanillo, Enrique J. Cobos

**Affiliations:** ^1^Department of Pharmacology, Faculty of Medicine, University of Granada, Granada, Spain; ^2^Institute of Neuroscience, The Biomedical Research Centre, University of Granada, Granada, Spain; ^3^Drug Discovery and Preclinical Development, ESTEVE, Barcelona, Spain; ^4^Teófilo Hernando Institute for Drug Discovery, Madrid, Spain; ^5^Biosanitary Research Institute, University Hospital Complex of Granada, Granada, Spain

**Keywords:** sigma-1 receptors, morphine, pain, analgesia, tolerance, joint inflammation, grip strength, von Frey threshold

## Abstract

Sigma-1 receptor antagonism increases the effects of morphine on nociceptive pain, even in morphine-tolerant animals. However, it is unknown whether these receptors are able to modulate morphine antinociception and tolerance during inflammatory pain. Here we used a mouse model to test the modulation of morphine effects by the selective sigma-1 antagonist S1RA (MR309), by determining its effect on inflammatory tactile allodynia (von Frey filaments) and on grip strength deficits induced by joint inflammation (a measure of pain-induced functional disability), and compared the results with those for nociceptive heat pain recorded with the unilateral hot plate (55°C) test. The subcutaneous (s.c.) administration of morphine induced antinociceptive effects to heat stimuli, and restored mechanical threshold and grip strength in mice with periarticular inflammation induced by Complete Freund’s Adjuvant. S1RA (80 mg/kg, s.c.) administered alone did not induce any effect on nociceptive heat pain or inflammatory allodynia, but was able to partially reverse grip strength deficits. The association of S1RA with morphine, at doses inducing little or no analgesic-like effects when administered alone, resulted in a marked antinociceptive effect to heat stimuli and complete reversion of inflammatory tactile allodynia. However, S1RA administration did not increase the effect of morphine on grip strength deficits induced by joint inflammation. When S1RA (80 mg/kg, s.c.) was administered to morphine-tolerant animals, it rescued the analgesic-like effects of this opioid in all three pain measures. However, when S1RA was repeatedly given during the induction of morphine tolerance (and not on the day of behavioral evaluation) it failed to affect tolerance to the effects of morphine on nociceptive heat pain or inflammatory allodynia, but completely preserved the effects of this opioid on grip strength deficits. These effects of S1RA on morphine tolerance cannot be explained by pharmacokinetic interactions, given that the administration of S1RA did not modify concentrations of morphine or morphine-3-glucuronide (a major morphine metabolite) in morphine-tolerant animals in plasma or brain tissue. We conclude that sigma-1 receptors play a pivotal role in the control of morphine analgesia and tolerance in nociceptive and inflammatory pain, although in a manner dependent on the type of painful stimulus explored.

## Introduction

Inflammatory pain is characterized by a decrease in the cutaneous sensory threshold, and by pain-induced decreases in physical function, which affect the quality of life of patients with inflammatory conditions (Salaffi et al., 2009). Cutaneous mechanical thresholds and physical function can be measured, in both humans and rodents, with von Frey filaments (cutaneous sensitivity) and grip strength testing (physical function) (Chandran et al., 2009; Cobos and Portillo-Salido, 2013; Lee, 2013; Helfert et al., 2015). Alterations in von Frey thresholds and pain-induced grip strength deficits during inflammation result (at least partially) from non-overlapping mechanisms, including the involvement of different subsets of primary afferents (Montilla-García et al., 2017). Despite their differences, both of these outcomes are sensitive to opioid analgesics, which can restore both normal sensory thresholds and physical functioning (Montilla-García et al., 2017).

Sigma-1 receptors are a promising novel pharmacological target for pain treatment (Romero et al., 2016; Sánchez-Fernández et al., 2017). Among the selective sigma-1 antagonists, the best characterized is S1RA, also known as MR309 (Vela et al., 2015). This compound is the only sigma-1 antagonist which has been evaluated in clinical trials with an intended indication for pain relief. S1RA has already been evaluated with positive results in several Phase II clinical trials for neuropathic pain (Bruna et al., 2018). Preclinical studies have shown that sigma-1 inhibition enhances the antinociception induced by opioid drugs, including morphine (reviewed in Zamanillo et al., 2013; Sánchez-Fernández et al., 2017). In addition, sigma-1 antagonism is able to rescue morphine antinociception in mice rendered tolerant to this opioid (Vidal-Torres et al., 2013; Rodríguez-Muñoz et al., 2015). Therefore, sigma-1 antagonists are promising tools as opioid adjuvants (Vela et al., 2015; Sánchez-Fernández et al., 2017), and in fact a further potential indication for S1RA in human patients might be to enhance opioid analgesia (Vela et al., 2015). Preclinical findings on the enhancement of opioid antinociception by sigma-1 inhibition have thus far been reported exclusively in models of nociceptive pain. It is known that opioid receptor functioning can change during inflammation (reviewed in Stein et al., 2009); therefore, the mechanisms involved in the modulation of opioid antinociception by sigma-1 receptor may not be the same during inflammation as in conditions not involving injury.

In light of these antecedents, we aimed to test whether the sigma-1 receptor antagonist S1RA enhanced morphine antinociception or modulated morphine analgesic tolerance during inflammatory pain. We measured both the antiallodynic effect of morphine as a measure of cutaneous sensory hypersensitivity, and the recovery of grip strength deficits induced by this opioid as a measure of the impact of pain on physical function. As a control for the known effects of sigma-1 antagonism on nociceptive pain, we also investigated heat antinociception in animals without inflammation. This allowed us to compare the effects of morphine, S1RA, and their association on nociceptive heat pain, inflammatory tactile allodynia and functional deficits (grip strength) induced by inflammatory pain.

## Materials and Methods

### Experimental Animals

Female CD1 mice (Charles River, Barcelona, Spain) were used in all experiments. We choose female animals because it has been reported that women may be at greater risk for pain-related disability than men (e.g., Unruh, 1996; Stubbs et al., 2010). Animals weighing 25–30 g were tested randomly throughout the estrous cycle. This mouse strain has been previously reported not to show variations in opioid analgesia during the phases of the estrous cycle (Mogil et al., 2000). All mice were housed in colony cages with free access to food and water prior to the experiments, and were kept in temperature- and light-controlled rooms (22 ± 2°C, 12-h light–dark cycle). The experiments were performed during the light phase from 9:00 a.m. to 3:00 p.m. Animal care was provided in accordance with institutional (Research Ethics Committee of the University of Granada, Granada, Spain), regional (Junta de Andalucía, Spain) and international standards (European Communities Council directive 2010/63). All mice were used in only one experimental procedure (heat nociception, von Frey testing or grip strength measurement).

### CFA-Induced Periarticular Inflammation

To induce the inflammation, mice were injected periarticularly with Complete Freund’s Adjuvant (CFA) (Sigma-Aldrich, Madrid, Spain) according to a previously described method (Montilla-García et al., 2017). In most experiments, CFA was administered subcutaneously (s.c.) around the tibiotarsal joint in two separate injections to the inner and outer side of the joint, at a volume of 15 μL/injection (30 μL/paw) to obtain homogeneous inflammation. In some experiments CFA was administered with the same procedure described above but using a lower injection volume (5 μL/injection; i.e., 10 μL/paw). Control animals received the same volume of sterile physiological saline (0.9% NaCl) with the same procedure. Injections were made with a 1710 TLL Hamilton microsyringe (Teknokroma, Barcelona, Spain) and a 30½-gauge needle under isoflurane anesthesia (IsoVet^®^, B. Braun, Barcelona, Spain). Behavioral evaluations in mice with induced inflammation were done 2 days after CFA or saline injection, since we previously reported that both tactile allodynia and grip strength deficits peak at this time (Montilla-García et al., 2017). Inflammatory edema was monitored by measuring ankle thickness with an electronic caliper (Montilla-García et al., 2017), also 2 days after CFA administration.

### Drugs and Drug Administration

We used the opioid agonist morphine (supplied by the General Directorate of Pharmacy and Drugs, Spanish Ministry of Health, Spain). S1RA (4-[2-[[5-methyl-1-(2-naphthalenyl)1H-pyraol-3-yl]oxy]ethyl] morpholine hydrochloride) (DC Chemicals, Shanghai, China) was used as a selective sigma-1 antagonist (Cobos et al., 2008; Romero et al., 2012). The dose of sigma-1 antagonist used in the present study (80 mg/kg) was high enough to induce a maximal effect in several pain models (Nieto et al., 2012; Sánchez-Fernández et al., 2013, 2014; Tejada et al., 2014, 2017). This same dose has been used in the formalin test (Romero et al., 2012), and higher doses of this compound (128 mg/kg) have been used in inflammatory heat hyperalgesia (Tejada et al., 2014, 2017), neuropathic cold allodynia (Nieto et al., 2012), and visceral pain (González-Cano et al., 2013). This last study showed that the s.c. administration of S1RA at 128 mg/kg still had selective analgesic effects (present in wild-type but absent in sigma-1 knockout mice). PRE-084 ([2-(4-morpholinethyl) 1-phenylcyclohexanecarboxylate hydrochloride]) (Tocris Cookson Ltd., Bristol, United Kingdom) was used as a selective sigma-1 agonist (Hayashi and Su, 2004; Cobos et al., 2008). We used half of the dose for PRE-084 than the dose used for S1RA in all experiments (i.e., 40 mg/kg of PRE-084). This dose was selected based in our previous experience in which we usually use this proportion of PRE-084/S1RA to fully reverse the effects of the sigma-1 antagonist (e.g., Sánchez-Fernández et al., 2013, 2014). In addition, in some experiments we used (+)-pentazocine (Sigma-Aldrich S.A.) as an additional selective sigma-1 agonist (Hayashi and Su, 2004; Cobos et al., 2008). All drugs were dissolved in sterile physiological saline (0.9% NaCl); the PRE-084 solution was appropriately alkalinized with NaOH. To evaluate the effects of systemic treatments, drugs were injected s.c. into the interscapular zone in a volume of 5 mL/kg. When the effect of the association of two or more drugs was tested, each drug was injected into a different area of the interscapular zone.

In experiments on the acute effects of systemic morphine alone or its association with S1RA, morphine was injected s.c. 30 min before the behavioral evaluation, and S1RA immediately before morphine injection. When PRE-084 or (+)-pentazocine were used to reverse the effect of S1RA, they were injected s.c. 5 min before S1RA.

Morphine tolerance was induced by a modification of the protocol we used in a previous study (Vidal-Torres et al., 2013). Briefly, morphine tolerance was induced with a 3-day cumulative dosage regimen consisting of twice-daily s.c. injections (b.i.d.) at 9:30 a.m. and 9:30 p.m., starting on day 1 at 9:30 a.m. The individual doses were 30 mg/kg (a.m.) and 45 mg/kg (p.m.) on day 1, 60 mg/kg (a.m.) and 80 mg/kg (p.m.) on day 2, and 100 mg/kg (a.m.) and 120 mg/kg (p.m.) on day 3. To avoid tissue lesions by repeated injections, morphine administration was rotated in each of the four quadrants of the back of the mice.

**FIGURE 1:**
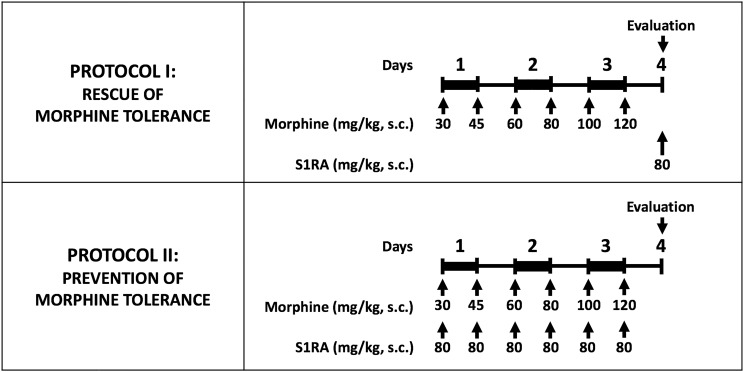
Experimental protocols used to investigate the effect of S1RA on morphine tolerance. Morphine tolerance was induced with a 3-day cumulative dosage regimen using the subcutaneous doses of morphine shown in the Figure. The upper panel shows the protocol used to test the effects of S1RA on rescue of the effect of morphine from tolerance once it was fully developed. The lower panel shows the protocol to study the effect of S1RA on prevention of the development of morphine tolerance. Drugs or their solvent (saline) were administered subcutaneously (s.c.). In all cases, “Evaluation” indicates the time when heat nociception, von Frey threshold or grip strength was measured, which was always on day 4 after the first morphine administration. On the evaluation day all animals received a dose of morphine (4 or 8 mg/kg sc. depending on the experiment; see text for details) 30 min before the pain response was evaluated.

To study whether S1RA administration was able to rescue morphine antinociception from tolerance once the latter was fully developed, on day 4, after the tolerance protocol was completed, mice were randomized to receive a test dose of morphine s.c. (4 mg/kg in tactile allodynia, and 8 mg/kg in heat nociception and grip strength) alone or associated with S1RA (80 mg/kg, s.c. for both tests), and then the behavioral effects were recorded (Figure 1, Protocol I). As in the protocol used to explore the acute effect of morphine alone and the influence of S1RA on the effects of this opioid, morphine was administered immediately after S1RA, 30 min before the behavioral evaluation. PRE-084 was administered 5 min before S1RA.

To study whether S1RA was able to prevent the development of morphine tolerance, mice were given S1RA (b.i.d. 80 mg/kg, s.c.) immediately after each morphine injection during the induction of analgesic tolerance (Figure 1, Protocol II). For each set of injections, each drug was injected in a different area of the interscapular zone according to the rotation protocol. Behavioral testing was done on day 4 with the same doses of morphine as reported in the preceding paragraph.

Injections of the drug solvent (saline) were used in all cases as a control.

### Measurement of Heat Nociception (Unilateral Hot Plate)

Heat nociception was assessed as previously described (Menéndez et al., 2002; Montilla-García et al., 2018). The plantar side of the stimulated hind paw was placed on the surface of a thermal analgesiometer (Model PE34, Series 8, IITC Life Science Inc., Los Angeles, CA, United States) previously set at 55 ± 1°C until the animal showed a paw withdrawal response. The latency in seconds from paw stimulation to the behavioral response was measured with a digital chronometer. Only a clear unilateral withdrawal of the paw was recorded as a nociceptive response. We avoided simultaneous heat stimulation in both hind paws by placing the plantar side of the tested hind paw on the hot plate while the other hind paw was placed on filter paper (off the hot plate) during observations (see Supplementary Video 2 in2 in Montilla-García et al., 2018). Two alternating evaluations were done in each hind paw at intervals of 1 min between each stimulation. A 50-s cut-off was used for each measurement to prevent tissue damage. The mean value of the two averaged measurements for each hind paw was used to analyze the effects of treatments.

### Measurement of von Frey Threshold

Tactile allodynia to a punctate stimulus was studied with the method described in a previous publication (Cobos et al., 2018). Briefly, animals were acclimated for 2 h in methacrylate test compartments (7.5 cm wide × 7.5 cm long × 15 cm high) placed on an elevated mesh-bottomed platform, to provide access to the plantar surface of the hind paws. The von Frey stimulations were applied to the heel, because our CFA injection protocol led to inflammation and tactile allodynia most prominently in this area (Montilla-García et al., 2017). A logarithmic series of calibrated von Frey monofilaments (Stoelting, Wood Dale, IL, United States), with bending forces that ranged from 0.02 to 1.4 g, were applied with the up–down paradigm (Chaplan et al., 1994), starting with the 0.6-g filament. Filaments were applied twice for 2–3 s, with between-application intervals of at least 30 s to avoid sensitization to the mechanical stimuli. The response to the filament was considered positive if immediate licking or biting, flinching or rapid withdrawal of the stimulated paw was observed.

### Measurement of Grip Strength

Grip strength was measured with a computerized grip strength meter (Model 47200, Ugo Basile, Varese, Italy) according to the method reported previously (Montilla-García et al., 2017). To measure grip strength in the hind paws, the experimenter held the mouse gently by the base of the tail, allowing the animal to grasp the metal bar of the grip strength meter with its hind paws. The metal bar was connected to a force transducer that automatically recorded the peak force of each measurement in grams (g). Hind limb grip strength in each mouse was measured in triplicate. To prevent mice from gripping the metal bar with their forepaws during the recording, the animals were first allowed to grasp a wire mesh cylinder with their forepaws (see Supplementary Video in Montilla-García et al., 2017). Baseline grip strength values were recorded for each animal as the average of two determinations on different days before the administration of CFA or saline. This value was considered 100% of grip strength and used as a reference for subsequent determinations.

### Determination of the Concentration of S1RA, Morphine and Morphine 3-Glucuronide in Plasma and Brain Tissue

To study whether the effects of S1RA on morphine tolerance were due to pharmacokinetic interactions between the sigma-1 antagonist and the opioid drug, we measured the concentration in plasma and brain tissue of S1RA, morphine and morphine-3-glucuronide, the major morphine metabolite in rodents (Pasternak and Pan, 2013). The concentrations of these compounds in plasma and brain tissue were measured according to the time when the behavioral effects of drug treatments were assessed, as described in the Section“Drugs and Drug Administration.” Briefly, a terminal blood sample was drawn from each mouse by cardiac puncture, at the appropriate time after vehicle or compound dosing. Blood samples were collected in heparinized tubes and centrifuged at 2,000 g for 10 min to obtain plasma. Immediately after blood extraction, whole brains were removed. Plasma samples and brains were stored at -80°C until analysis. Each brain was weighed and homogenized in 4 mL Dulbecco’s phosphate buffered saline immediately before drug concentrations were determined. Protein was precipitated with acetonitrile, and samples were analyzed by high-performance liquid chromatography–triple quadrupole mass spectrometry (HPLC-MS/MS). The concentrations of compounds in plasma and brain were determined by least-squares linear regression with a calibration curve.

### Data Analysis

The data were analyzed with the SigmaPlot 12.0 program (Systat Software Inc., San Jose, CA, United States). One-way, two-way, or two-way repeated-measures analysis of variance (ANOVA) was used depending on the experiment; Student–Newman–Keuls *post hoc* test was done in all cases. The differences between means were considered significant when the *P*-value was below 0.05.

## Results

### Modulation by S1RA of Morphine-Induced Antinociception to Heat Stimulus

**FIGURE 2:**
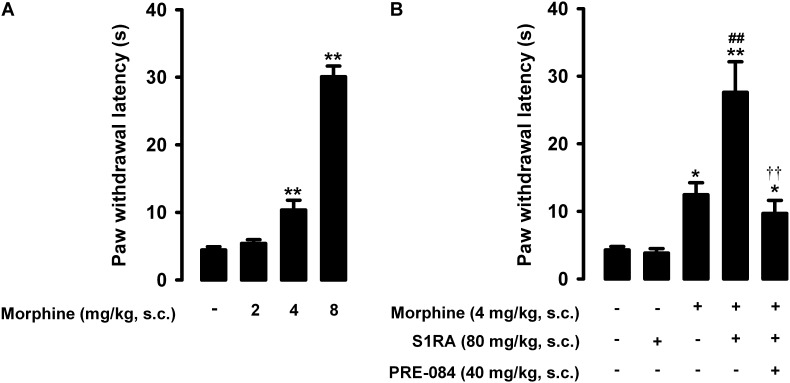
Effects of morphine alone or associated with S1RA on nociceptive pain induced by heat stimulation. The results represent paw withdrawal latency during stimulation of the hind paw at 55°C. **(A)** Effect of the subcutaneous (s.c.) administration of different doses of morphine (2–8 mg/kg) or its vehicle. **(B)** Effect of the s.c. administration of morphine (4 mg/kg), S1RA (80 mg/kg) or their vehicle; morphine (4 mg/kg) + S1RA (80 mg/kg), and the association of these drugs with PRE-084 (40 mg/kg) or its vehicle. Each bar and vertical line represent the mean ± SEM of values obtained in 8–10 animals. **(A)** Statistically significant differences between the values obtained in animals treated with morphine or its vehicle: ^∗∗^*P* < 0.01 (one-way ANOVA followed by Student–Newman–Keuls test). **(B)** Statistically significant differences between the values obtained in animals treated with: morphine or its vehicle (^∗^*P* < 0.05, ^∗∗^*P* < 0.01); morphine + S1RA or its vehicle (^##^*P* < 0.01); and morphine + S1RA associated with PRE-084 or its vehicle (*P* < 0.01) (one-way ANOVA followed by Student–Newman–Keuls test).

The paw withdrawal latency to a nociceptive heat stimulus in mice without inflammation was short, i.e., less than 5s (Figure 2A). Morphine administration (2–8 mg/kg, s.c.) induced dose-dependent robust antinociceptive effects, reaching values of about 30 s at the highest dose tested (Figure 2A).

In contrast to morphine, the selective sigma-1 antagonist S1RA (80 mg/kg, s.c.) failed to alter the response latency of mice to a nociceptive heat stimulus (Figure 2B). However, when we associated this dose of S1RA with morphine 4 mg/kg (s.c.), the response latency increased markedly (Figure 2B). S1RA also increased the antinociceptive effect induced by morphine 2 mg/kg (s.c.), but to a lesser extent (data not shown). We also evaluated the effects of the sigma-1 agonist PRE-084 (40 mg/kg, s.c.) on heat antinociception induced by the association of S1RA with morphine, and found that treatment with the sigma-1 agonist abolished the S1RA-induced potentiation of morphine antinociception (Figure 2B). When PRE-084 was administered alone it failed to induce any effect on nociception (data not shown). These results support the selectivity of the effects induced by S1RA. Therefore, S1RA appeared to enhance the antinociceptive effect of morphine to a heat stimulus through sigma-1 inhibition. These results are summarized in Table 1.

**Table 1:** Summary of the main results for the role of sigma-1 receptors in nociceptive heat pain and inflammation-induced tactile allodynia and grip strength deficits.

Type of pain	Stimulus	Sensitivity to morphine	Sensitivity to S1RA	Potentiation of morphine by S1RA	Effect of S1RA on morphine tolerance	
					Rescue of morphine tolerance	Prevention of morphine tolerance
Nociceptive	Heat	+	-	+	+	-
Inflammatory	von Frey	+++	-	+	+	-
	Grip strength	++	+	-	+	+


*S1RA was always administered subcutaneously (s.c.) at 80 mg/kg. The doses of morphine used to test the potentiation of its effect by S1RA varied according to the sensitivity to the opioid in each test (1 mg/kg for von Frey testing in mice with inflammation, and 2–4 mg/kg for nociceptive heat pain and grip strength deficits induced by inflammation), and in the evaluation of analgesic tolerance (4 mg/kg for von Frey testing in mice with inflammation, and 8 mg/kg for nociceptive heat pain and grip strength deficits induced by inflammation)*.

### Modulation by S1RA of Tolerance to the Antinociceptive Effect of Morphine in Response to Heat Stimulus

**FIGURE 3:**
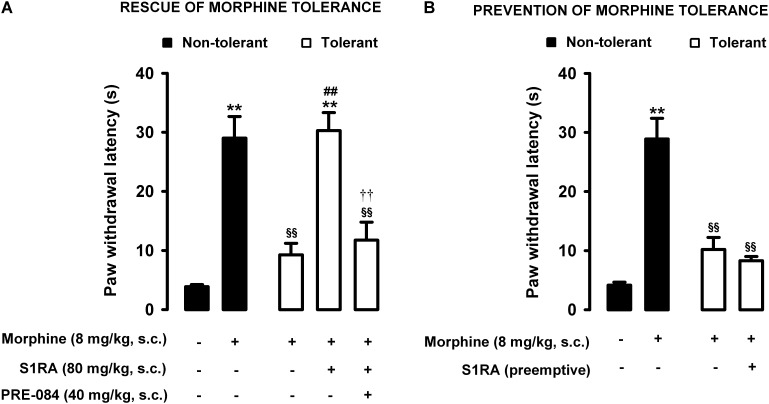
Modulation by S1RA of tolerance to the effect of morphine on nociceptive pain caused by heat stimulation. The results represent paw withdrawal latency during stimulation of the hind paw at 55°C. **(A)** Rescue of morphine tolerance by S1RA: animals were repeatedly treated during 3 days with morphine (tolerant, white bars) or its vehicle (non-tolerant, black bars), according to Protocol I in Figure 1. On the day of evaluation (day 4) mice were treated subcutaneously (s.c.) with morphine (8 mg/kg) or its vehicle, morphine (8 mg/kg) + S1RA (80 mg/kg) or its vehicle, or the combination of these drugs with PRE-084 (40 mg/kg) or its vehicle. **(B)** Prevention of morphine tolerance by S1RA: animals were treated s.c. with S1RA (80 mg/kg) or its vehicle immediately before each dose of morphine (tolerant, white bars) during the induction of morphine tolerance, according to Protocol II in Figure 1. Control mice (non-tolerant, black bars) received the vehicle of morphine and S1RA during 3 days, according to Protocol II in Figure 1. On the day of evaluation (day 4) tolerant and non-tolerant mice were treated with morphine only (8 mg/kg, s.c.) or its vehicle. Each bar and vertical line represent the mean ± SEM of the values obtained in 8–10 animals. **(A,B)** Statistically significant differences between the values obtained in mice treated with morphine or its vehicle (^∗∗^*P* < 0.01), and between the values obtained in tolerant and non-tolerant animals treated with morphine on the day of the evaluation (^§§^ *P* < 0.01) (one-way ANOVA followed by Student–Newman–Keuls test). **(A)** Statistically significant differences in tolerant mice between the values obtained in mice treated on the day of evaluation with: morphine + S1RA or its vehicle (^##^*P* < 0.01); morphine + S1RA associated with PRE-084 or its vehicle (*P* < 0.01) (one-way ANOVA followed by Student–Newman–Keuls test). **(B)** There were no statistically significant differences in the effect of morphine on the day of evaluation between the values in mice treated with S1RA or its vehicle during the induction of morphine tolerance (two-way ANOVA followed by Student–Newman–Keuls test).

Animals were rendered morphine-tolerant with a 3-day escalating dosage regimen (Figure 1). Control non-tolerant mice were treated with the morphine vehicle. On day 4, non-tolerant mice showed a marked increase in the response latency induced by a morphine dose shown in the previous experiments (see section “Modulation by S1RA of Morphine-Induced Antinociception to Heat Stimulus”) to induce evident antinociception (8 mg/kg, s.c.) (Figure 3A, black bars). However, the effect induced by this morphine dose was markedly lower in morphine-tolerant mice, with a paw withdrawal latency of 29.01 ± 3.67 s in non-tolerant mice vs. 9.25 ± 1.95 s in tolerant mice in response to morphine (Figure 3A). In animals rendered tolerant to morphine, we associated the administration of S1RA (80 mg/kg, s.c.) to morphine (8 mg/kg, s.c.) according to Protocol I in Figure 1, and found that the response latency was longer, reaching times similar to those in control non-tolerant mice. This result indicated that S1RA was able to rescue morphine antinociception in tolerant animals (Figure 3A). The administration of PRE-084 (40 mg/kg, s.c.) completely abolished the increase in the antinociceptive effect of morphine induced by S1RA in morphine-tolerant mice: the latency values were close to those found in tolerant mice treated with morphine alone on the day of the experiment (Figure 3A). When PRE-084 was administered alone it failed to induce any effect on nociception (data not shown).

We also tested whether the repeated administration of S1RA (80 mg/kg, s.c.) before each dose of morphine during the 3-day period of morphine administration had any pre-emptive effect on the appearance of morphine tolerance to its effect on nociceptive heat pain (according to Protocol II in Figure 1). When the effect of morphine (8 mg/kg, s.c.) was tested on day 4 without any further administration of S1RA, the effect of morphine was lower (Figure 3B). These results indicated that tolerance to the antinociceptive effect of morphine on the response to a heat stimulus was present despite repeated pre-emptive S1RA administration.

Therefore, sigma-1 receptor inhibition by S1RA was able to restore the antinociceptive effect of morphine to heat stimulation in mice tolerant to this opioid, but pre-emptive S1RA treatment failed to affect the development of tolerance to the effect of morphine on nociceptive heat pain. These results are summarized in Table 1.

### Modulation by S1RA of the Antiallodynic Effect of Morphine During Inflammation

**FIGURE 4:**
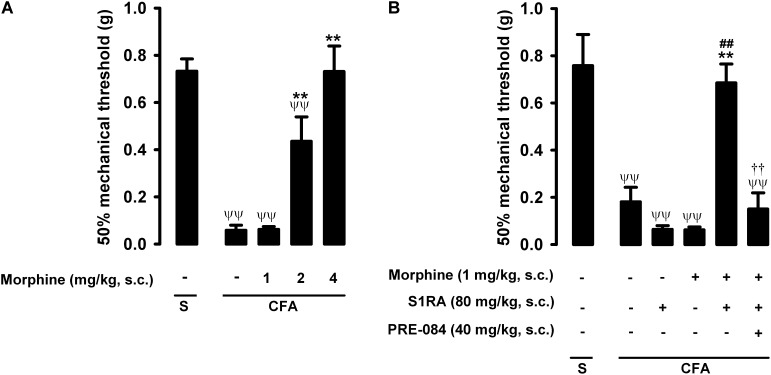
Effects of morphine alone or associated with S1RA on inflammatory mechanical allodynia. The results represent the 50% mechanical threshold (determined with von Frey filaments) in mice treated periarticularly (30 μL/paw) with CFA or saline (S). **(A)** Effect of the subcutaneous (s.c.) administration of different doses of morphine (1–4 mg/kg) or its vehicle. **(B)** Effect of the s.c. administration of morphine (1 mg/kg), S1RA (80 mg/kg) or its vehicle; morphine (1 mg/kg) + S1RA (80 mg/kg), and the association of these drugs with PRE-084 (40 mg/kg) or its vehicle. Each bar and vertical line represent the mean ± SEM of values obtained in 8–10 animals. **(A,B)** Statistically significant differences between the values obtained in: animals with and without inflammation (^ψψ^*P* < 0.01); animals treated with morphine or its vehicle (^∗∗^*P* < 0.01) (one-way ANOVA followed by Student–Newman–Keuls test). **(B)** Statistically significant differences between the values obtained in animals with inflammation treated with: morphine + S1RA or its vehicle (^##^*P* < 0.01); morphine + S1RA associated with PRE-084 or its vehicle. ^#^ (*P* < 0.01) (one-way ANOVA followed by Student–Newman–Keuls test).

In mice with CFA injected around the tibiotarsal joint (30 μL/paw), the mechanical pain threshold in the heel was markedly reduced, denoting the presence of tactile allodynia (0.73 ± 0.05 g and 0.06 ± 0.01 g in mice without and with inflammation, respectively) (Figure 4A). Joint inflammation did not induce alterations in the von Frey threshold in the non-inflamed area (pad) of the paw (data not shown), indicating that sensory alterations appeared to be restricted to the inflamed area. Morphine administration (1–4 mg/kg, s.c.) induced a dose-dependent antiallodynic effect in animals with inflammation, and the normal mechanical threshold was fully recovered at the highest dose tested (Figure 4A). The effect of morphine was more prominent in tactile allodynia than in nociceptive heat pain: the doses needed to induce significant effects were lower in the former assay (compare Figure 2A, 4A).

The administration of S1RA alone (80 mg/kg, s.c.) did not ameliorate inflammatory tactile allodynia (Figure 4B). However, the association of this dose of S1RA with a low dose of morphine (1 mg/kg, s.c.), which was also devoid of antiallodynic effect *per se*, resulted in a marked synergistic increase in the mechanical threshold in mice with induced inflammation: the values in these animals were similar to those in mice in which inflammation was not induced (Figure 4B). As reported above for heat nociception, the administration of the sigma-1 agonist PRE-084 (40 mg/kg, s.c.) abolished the potentiation induced by S1RA of the antiallodynic effect of morphine (Figure 4B); this result supports the notion that the effects induced by S1RA are selective. We thus found that S1RA enhanced the antiallodynic effect of morphine through sigma-1 inhibition. These results are summarized in Table 1.

Interestingly, when this dose of S1RA (80 mg/kg, s.c.) was assayed in mice with a lower degree of inflammation (10 μL CFA/paw), it was able to fully recover the normal mechanical threshold in mice with inflammation in the absence of morphine administration (Supplementary Figure S1), indicating that this compound is able to exert antiallodynic effects, albeit in milder inflammation. The antiallodynic effect induced by S1RA was abolished by PRE-084 (40 mg/kg, s.c.), a result that supports the selectivity of the effects induced by S1RA (Supplementary Figure S1).

### Modulation by S1RA of Tolerance to the Antiallodynic Effect of Morphine During Inflammation

**FIGURE 5:**
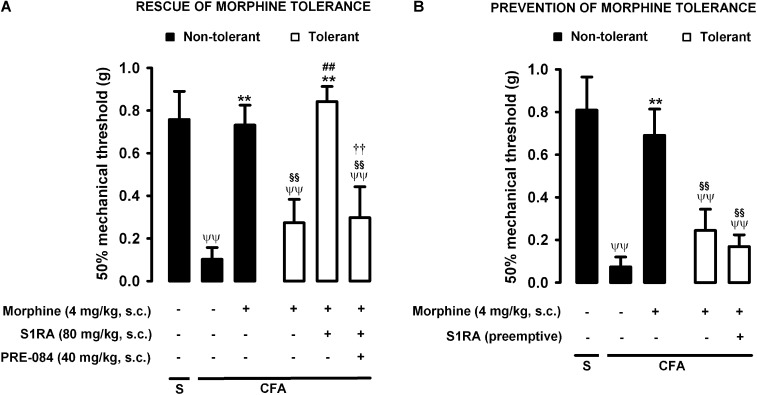
Modulation by S1RA of tolerance to the effect of morphine on inflammatory mechanical allodynia. The results represent the 50% mechanical threshold (determined with von Frey filaments) in mice treated periarticularly (30 μL/paw) with CFA or saline (S). **(A)** Rescue of morphine tolerance by S1RA: Animals were repeatedly treated during 3 days with morphine (tolerant, white bars) or its vehicle (non-tolerant, black bars), according to Protocol I in Figure 1. On the day of evaluation (day 4) mice were treated subcutaneously (s.c.) with morphine (4 mg/kg) or its vehicle, the combination of morphine (4 mg/kg) and S1RA (80 mg/kg) or its vehicle, and the combination of these drugs with PRE-084 (40 mg/kg) or its vehicle. **(B)** Prevention of morphine tolerance by S1RA: Animals were treated s.c. with S1RA (80 mg/kg) or its vehicle immediately before each dose of morphine (tolerant, white bars) during the induction of morphine tolerance, according to Protocol II in Figure 1. Control mice (non-tolerant, black bars) received the vehicles of morphine or S1RA during 3 days, according to Protocol II in Figure 1. On the day of evaluation (day 4), tolerant and non-tolerant mice were treated s.c. with morphine only (4 mg/kg) or its vehicle. Each bar and vertical line represent the mean ± SEM of the values obtained in 8–10 animals. **(A,B)** Statistically significant differences between the values obtained in: mice with and without inflammation that received the same treatment (^ψψ^*P* < 0.01); tolerant and non-tolerant animals treated with morphine on the day of the evaluation (^§§^ *P* < 0.01); mice treated with saline or morphine on the day of evaluation (^∗∗^*P* < 0.01) (one-way ANOVA followed by Student–Newman–Keuls test). **(A)** Statistically significant differences in tolerant mice between the values obtained in animals treated on the day of evaluation with: morphine + S1RA or its vehicle (^##^*P* < 0.01); morphine + S1RA associated with PRE-084 or its vehicle (P < 0.01) (one-way ANOVA followed by Student–Newman–Keuls test). **(B)** There were no statistically significant differences in the effect of morphine on the day of evaluation between mice treated with S1RA or its vehicle during the induction of morphine tolerance (two-way ANOVA followed by Student–Newman–Keuls test).

As described above for heat nociception, animals were rendered morphine-tolerant with a 3-day escalating dosage regimen (Figure 1), whereas control non-tolerant mice received the morphine vehicle. On day 4, non-tolerant mice with inflammation (30 μL CFA/paw) showed complete reversion of inflammatory tactile allodynia induced by the acute administration of morphine 4 mg/kg (s.c.) (Figure 5A, black bars). However, in morphine-tolerant mice the antiallodynic effect induced by the same morphine dose was markedly lower, with a mechanical threshold of 0.73 ± 0.09 g in non-tolerant mice vs. 0.27 ± 0.11 g in tolerant mice with inflammation in response to morphine (Figure 5A). In animals rendered tolerant to morphine, we associated the administration of S1RA (80 mg/kg, s.c.) to morphine (4 mg/kg, s.c.) according to Protocol I in Figure 1, and found that the mechanical threshold was higher and similar to that found in non-tolerant mice with inflammation treated with this opioid drug (Figure 5A). These results indicate that S1RA was able to rescue the antiallodynic effect of morphine in tolerant animals. The administration of PRE-084 (40 mg/kg, s.c.) completely abolished the effect of S1RA in morphine-treated tolerant mice, yielding values close to those found in tolerant mice treated with morphine alone on the day of the experiment (Figure 5A).

We also tested whether the repeated administration of S1RA (80 mg/kg, s.c.) before each dose of morphine during the 3-day regimen of morphine administration had a pre-emptive effect on the development of morphine tolerance to its antiallodynic effect (according to Protocol II in Figure 1). When the effect of morphine (4 mg/kg, s.c.) was tested on day 4 without any further administration of S1RA, the effect of morphine was reduced (Figure 5B). These results indicate that tolerance to the antiallodynic effect of morphine appeared despite the repeated pre-emptive administration of S1RA.

Therefore, as in the results for heat nociception, sigma-1 receptor inhibition by S1RA was able to restore the morphine-induced antiallodynic effects in mice with inflammation tolerant to this opioid, but pre-emptive treatment with S1RA failed to affect the development of tolerance to the antiallodynic effect of morphine. These results are summarized in Table 1.

### Absence of Modulation by S1RA of Morphine-Induced Recovery of Grip Strength Deficits During Inflammation

**FIGURE 6:**
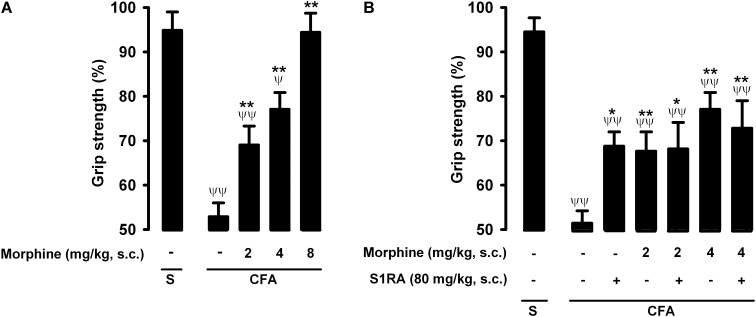
Effects of morphine alone or associated with S1RA on grip strength deficits induced by inflammation. The results represent the grip strength values, expressed as the average percentage of the baseline value in each individual mouse before the periarticular injection (30 μL/paw) with CFA or saline (S), **(A)** effect of the subcutaneous (s.c.) administration of different doses of morphine (2–8 mg/kg) or its vehicle. **(B)** Effect of the s.c. administration of morphine (2 or 4 mg/kg), S1RA (80 mg/kg) or its vehicle, and the association of morphine (2 or 4 mg/kg) with S1RA (80 mg/kg). Each bar and vertical line represent the mean ± SEM of values obtained in 8–10 animals. **(A,B)** Statistically significant differences between the values obtained in mice with and without inflammation: ^ψ^*P* < 0.05, ^ψψ^*P* < 0.01; and between animals with inflammation treated with morphine or its vehicle: ^∗^*P* < 0.05, ^∗∗^*P* < 0.01 (one-way ANOVA followed by Student–Newman–Keuls test). **(B)** There were no statistically significant differences between the values obtained in mice treated with morphine + S1RA or its vehicle (one-way ANOVA followed by Student–Newman–Keuls test).

Mice in which saline was injected periarticularly (30 μL/paw) showed grip strength values close to 100% of their baseline values, whereas in mice with joint inflammation induced by the injection of the same volume of CFA, grip strength was reduced to about half of its baseline value (Figure 6A, first two bars). Morphine administration (2–8 mg/kg, s.c.) induced a dose-dependent recovery of grip strength deficits in animals with inflammation, and the highest dose tested led to full recovery of normal grip strength values (Figure 6A). Morphine was less potent in reversing grip strength deficits than in inhibiting tactile allodynia during inflammation, and both endpoints were more sensitive to morphine than nociceptive heat pain (compare Figure 2A, 4A, 6A).

The administration of S1RA alone (80 mg/kg, s.c.) induced a slight but significant increase in grip strength values in mice with inflammation (Figure 6B). In contrast to the results for heat nociception and inflammatory tactile allodynia, S1RA administration did not enhance the effect of morphine at either 2 or 4 mg/kg (s.c.) (Figure 6B). To further confirm the lack of effect of S1RA on the effect of morphine on grip strength in mice with inflammation, we performed a time-course study of the drug effects. The association of S1RA with morphine did not alter the effect of this opioid drug administered at 2 mg/kg (s.c.) at any time-point tested between 30 and 180 min after drug administration (Supplementary Figure S2A). Similar results were found when we tested the effects of the association of S1RA with morphine 4 mg/kg (s.c.) (Supplementary Figure S2B).

Therefore, in contrast to the potentiation by S1RA of morphine-induced heat antinociception and the antiallodynic effects in mice with inflammation, S1RA was unable to modify the effects of morphine on grip strength deficits induced by joint inflammation. These results are summarized in Table 1.

### Modulation by S1RA of Tolerance to Morphine-Induced Recovery of Grip Strength Deficits During Inflammation

**FIGURE 7:**
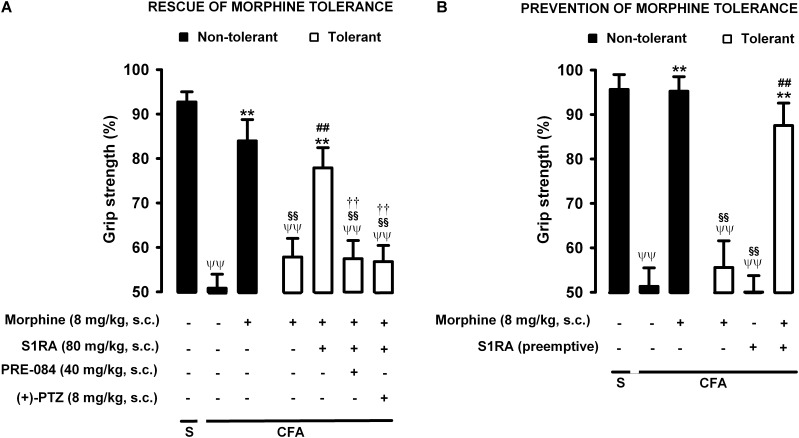
Modulation by S1RA of tolerance to the effect of morphine on grip strength deficits induced by inflammation. The results represent the grip strength values, expressed as the average percentage of the baseline value in each individual mouse before the periarticular injection, (30 μL/paw) with CFA or saline (S). **(A)** Rescue of morphine tolerance by S1RA: Animals were repeatedly treated subcutaneously (s.c.) during 3 days with morphine (tolerant, white bars) or its vehicle (non-tolerant, black bars), according to Protocol I in Figure 1. On the day of evaluation (day 4) mice were treated s.c. with morphine (8 mg/kg) or its vehicle; morphine (8 mg/kg) + S1RA (80 mg/kg) or its vehicle, and the combination of these drugs with PRE-084 (40 mg/kg), (+)-pentazocine [(+)-PTZ] or their vehicle. **(B)** Prevention of morphine tolerance by S1RA: animals were treated s.c. with S1RA (80 mg/kg) or its vehicle immediately before each dose of morphine (tolerant, white bars) during the induction of morphine tolerance, according to Protocol II in Figure 1. Control mice (non-tolerant, black bars) received the vehicle of morphine or S1RA during 3 days, according to Protocol II in Figure 1. On the day of evaluation (day 4), tolerant and non-tolerant mice were treated with morphine only (8 mg/kg, s.c.) or its vehicle. Each bar and vertical line represent the mean ± SEM of the values obtained in 8–10 animals. **(A,B)** Statistically significant differences between the values obtained in: mice with and without inflammation that received the same treatment (^ψψ^*P* < 0.01); tolerant and non-tolerant animals treated with morphine on the day of the experiment (^§§^ *P* < 0.01); mice treated with saline or morphine on the day of behavioral testing (^∗∗^*P* < 0.01); tolerant mice treated with S1RA or its vehicle either on the day of the evaluation or as pre-emptive treatment (^##^*P* < 0.01) (one-way ANOVA followed by Student–Newman–Keuls test). **(A)** Statistically significant differences between the values obtained in tolerant mice treated on the day of evaluation with morphine + S1RA associated with PRE-084, (+)-PTZ or their solvent: ^#^ *P* < 0.01 (two-way ANOVA followed by Student–Newman–Keuls test).

Animals were rendered morphine-tolerant by the same procedure described in previous sections (Figure 1), whereas control non-tolerant mice received morphine vehicle. In non-tolerant mice with inflammation (30 μL CFA/paw), grip strength deficits were markedly reversed in response to the acute administration of morphine 8 mg/kg (s.c.) (Figure 7A, black bars). However, the same morphine dose (8 mg/kg, s.c.) in morphine-tolerant mice had no significant effect on grip strength, which was about half of the baseline value (83.95 ± 4.8% in non-tolerant mice vs. 57.81 ± 4.2% in tolerant mice with inflammation in response to morphine) (Figure 7A). In animals rendered tolerant to morphine, we associated the administration of S1RA (80 mg/kg, s.c.) to morphine (8 mg/kg, s.c.) according to Protocol I in Figure 1, and found that grip strength reached values close to 80% of baseline measurements (Figure 7A). The administration of PRE-084 (40 mg/kg, s.c.) or (+)-pentazocine (8 mg/kg, s.c.) completely abolished the effect of the association of S1RA + morphine administered to morphine-treated tolerant mice, with values close to those in tolerant mice treated with morphine alone the day of the experiment (Figure 7A). These results support the selectivity of S1RA-induced effects.

We also tested whether the repeated administration of S1RA (80 mg/kg, s.c.) during the 3-day regimen of morphine administration (according to Protocol II in Figure 1) had a pre-emptive effect on the development of morphine tolerance to its effect on grip strength deficits. The repeated administration of S1RA, when mice were evaluated the day after the last S1RA administration, did not affect grip strength in morphine-tolerant mice with inflammation. However, when morphine (8 mg/kg, s.c.) was administered to these mice on the evaluation day, the effect in response to the opioid was robust (Figure 7B). These results indicate that S1RA was able to prevent morphine tolerance in this particular outcome, in contrast to the results for nociceptive heat pain and inflammatory tactile allodynia reported in the preceding sections.

Therefore the administration of S1RA, either when morphine tolerance was fully developed or during the induction of tolerance, was able to preserve the effect of morphine on grip strength deficits in mice with joint inflammation. These results are summarized in Table 1.

### Morphine, S1RA and PRE-084 do Not Alter Normal Grip Strength or CFA-Induced Inflammatory Edema

**FIGURE 8:**
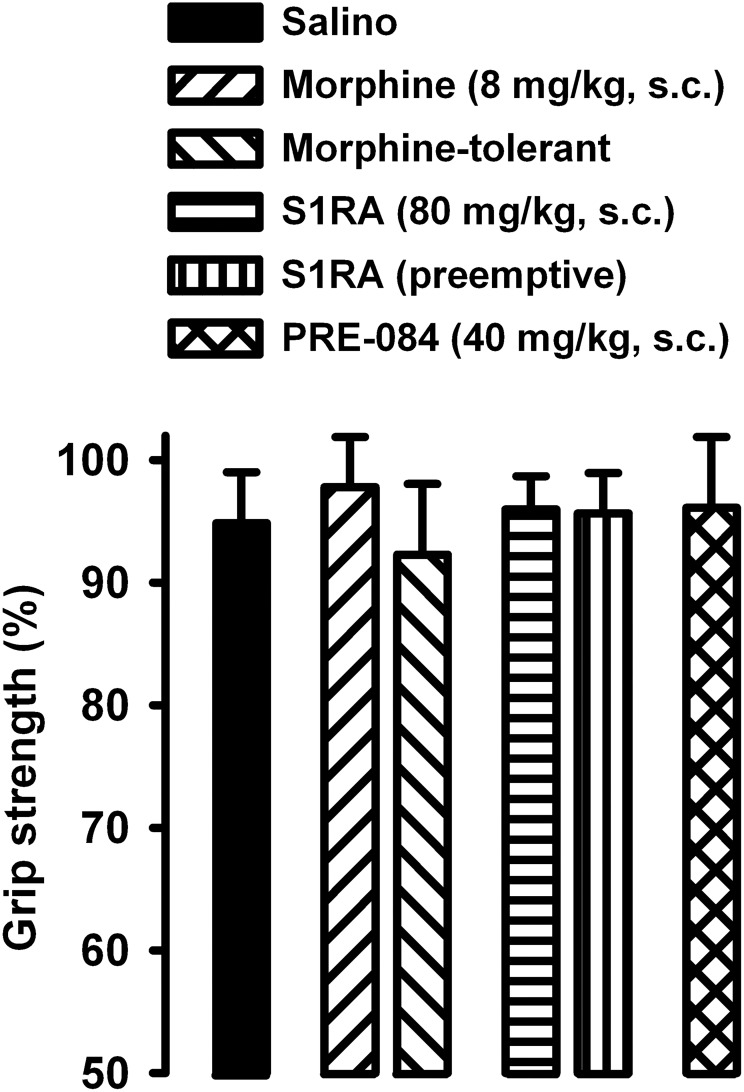
Absence of effects of morphine, S1RA and PRE-084 on grip strength in mice without inflammation. The results represent the grip strength values (expressed as the average percentage of the baseline value in each individual mouse before drug administration). Animals were treated subcutaneously (s.c.) with morphine (8 mg/kg,), S1RA (80 mg/kg) or PRE-084 (40 mg/kg) on the day of the experiment, or treated twice daily with morphine at escalating doses or with S1RA (80 mg/kg) during the 3 days before the behavioral evaluation (see section “Materials and Methods” for details). Each bar and vertical line represent the mean ± SEM of the values obtained in 8 animals. There were no statistically significant differences between values from untreated animals and drug-treated mice (one-way ANOVA).

We also tested whether the drugs used in the present study altered grip strength in mice without inflammation. The doses of morphine or S1RA (administered acutely or repeatedly) used in this study, as well as the acute administration of PRE-084 (at the dose used in our study), did not alter normal grip strength in animals without inflammation (Figure 8). These results suggest that the effects on grip strength in mice with inflammation, reported in the preceding section, were not due to the alteration of normal motor function.

**FIGURE 9:**
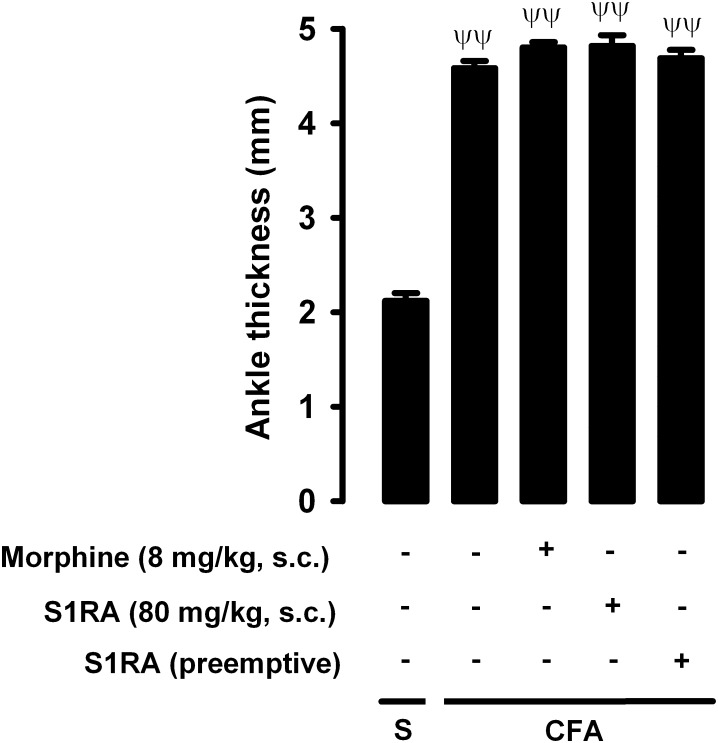
Effects of morphine and S1RA on ankle thickness in mice with inflammation. The results represent ankle thickness after the administration of CFA or saline (S). Animals were treated subcutaneously (s.c.) with morphine (8 mg/kg,) or S1RA (80 mg/kg) on the day of the experiment, or treated twice daily with S1RA (80 mg/kg) during the 3 days before the evaluation (see section “Materials and Methods” for details). Each bar and vertical line represent the mean ± SEM of the values obtained in 8 animals. The differences between the values from mice without or with inflammation were statistically significant (^ΨΨ^*P* < 0.01). There were no statistically significant differences between the values from mice with inflammation treated with the drugs and untreated mice with inflammation (one-way ANOVA followed by Student–Newman–Keuls test).

We also tested whether the drugs used in the present study reduced inflammatory edema, by measuring changes in ankle thickness in response to CFA administration. CFA injection produced a marked increase in ankle thickness in comparison to saline-treated mice, and this increase was not significantly altered by either the acute administration of morphine (8 mg/kg, s.c.) or the acute or repeated administration of S1RA (80 mg/kg, s.c.) (Figure 9). Therefore, none of these drugs appeared to have an antiedematous effect on CFA-induced inflammation.

### Pharmacokinetic Interaction Between S1RA and Morphine Does Not Affect Their Concentrations in Plasma or Brain Tissue

**FIGURE 10:**
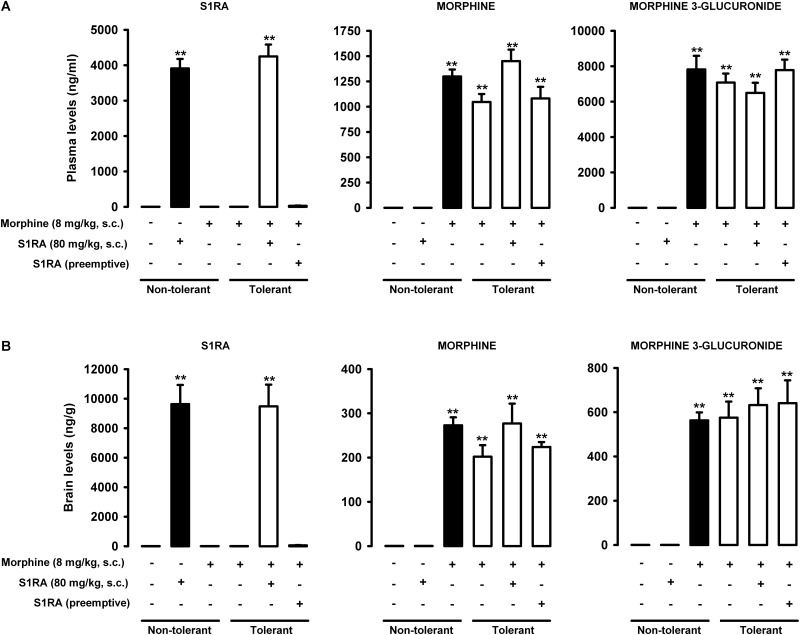
S1RA and morphine did not interact pharmacokinetically in a way that affected their concentrations in plasma or brain tissue. The levels of S1RA, morphine and morphine-3-glucuronide were measured by ultra-high performance liquid chromatography and tandem mass spectrometry in plasma **(A)** and brain homogenates **(B)**. Animals were repeatedly treated subcutaneously (s.c.) during 3 days with morphine (tolerant, white bars) or its vehicle (non-tolerant, black bars). On the day of sample collection (day 4) mice were treated s.c. with morphine (8 mg/kg) or its vehicle. S1RA (80 mg/kg, s.c.) was administered acutely on the day of sample collection (according to Protocol 1 in1 in Figure 1), or immediately before each dose of morphine during the induction of morphine tolerance (according to Protocol II in Figure 1). Each bar and vertical line represent the mean ± SEM of the values obtained in 5 animals. Statistically significant differences between the concentrations of S1RA, morphine or morphine-3-glucuronide in vehicle-treated animals and the rest of the experimental groups tested (^∗∗^*P* < 0.01). There were no statistically significant differences between the levels of S1RA when administered on the day of sample collection to morphine tolerant and non-tolerant animals; the levels of morphine when administered on the day of sample collection to morphine tolerant and non-tolerant animals, or between the levels of morphine-3-glucuronide when morphine was administered on the day of sample collection to morphine tolerant and non-tolerant animals (two-way ANOVA followed by Student–Newman–Keuls test).

After a single administration of S1RA (80 mg/kg, s.c.), the concentration of this sigma-1 antagonist was higher in brain tissue than in plasma (compare black bars in the left panels of Figure 10A,B). The repeated administration of morphine according to our protocol for the induction of tolerance did not alter the concentration of acutely administered S1RA (80 mg/kg, s.c., according to Protocol I in Figure 1) in either plasma or brain tissue (compare black and white bars in the left panels of Figure 10A,B). When we measured the concentration of S1RA after its repeated administration (80 mg/kg, s.c.) during the 3-day regimen of morphine administration, without any further S1RA injection on the day of plasma and brain collection (according to Protocol II in Figure 1), we observed no appreciable levels of this sigma-1 antagonist in any sample analyzed (see far-right bars in the left panels of Figure 10A,B for plasma and brain levels, respectively). Therefore, when S1RA was administered pre-emptively during the induction of morphine tolerance, there was no remaining S1RA in plasma or brain tissue at the time of the behavioral evaluations.

Morphine levels were higher in plasma than in brain tissue after the acute administration (8 mg/kg, s.c.) of this opioid (compare black bars in the middle panels of Figure 10A,B). Repeated morphine administration according to our protocol for the induction of morphine tolerance did not change morphine levels after the administration of this opioid on the day of sample collection in either plasma or brain tissue (compare black bar and the first white bar in the middle panels of Figure 10A,B). The acute or repeated administration of S1RA (80 mg/kg, s.c. according to Protocol I or II in Figure 1) did not alter morphine levels in either plasma or brain tissue in morphine-tolerant animals (see white bars in the middle panels of Figure 10A,B).

Like morphine, the concentration of morphine-3-glucuronide was higher in plasma than in brain tissue (compare black bars in the right panels of Figure 10A,B). This morphine metabolite was more abundant than the parent compound in both plasma and brain tissue after acute morphine administration (compare black bars in the middle and right panels of Figure 10A,B for plasma and brain tissue determinations, respectively). Repeated morphine administration according to our protocol to induce tolerance did not alter morphine-3-glucuronide levels in either plasma or brain samples after the administration of morphine on the day of the behavioral evaluation (compare black bar and the first white bar in the right panels of Figure 10A,B). The acute or repeated administration of S1RA (80 mg/kg, s.c.) did not alter morphine-3-glucuronide levels in morphine-tolerant animals in either plasma or brain tissue (white bars in the right panels of Figure 10A,B).

## Discussion

We compared the effects of morphine, S1RA and their association on three different pain measures: nociceptive heat pain, inflammatory tactile allodynia, and grip strength deficits induced by inflammation. In addition, we studied the effects of S1RA on morphine tolerance in these three different measures.

Our findings show that morphine was able to induce analgesic-like effects on nociceptive heat pain, inflammatory tactile allodynia and grip strength deficits induced by inflammation. However, the sensitivity to this opioid drug varied depending on the endpoint examined. Inflammatory allodynia and grip strength deficits induced by inflammation were more sensitive to the effects of morphine than nociceptive heat pain. These results are consistent with the widely reported increase in the effects of opioids on cutaneous sensory hypersensitivity during inflammation (reviewed in Stein et al., 2009), and indicate that the enhancement of opioid effects during inflammation, as observed in von Frey filament thresholds, is also evident in the functional deficit associated with this pathological condition. In previous work we compared the pharmacological characteristics of grip strength deficits and tactile allodynia during inflammation using several drugs from different pharmacological groups, including opioids (oxycodone and tramadol), non-steroidal anti-inflammatory drugs (ibuprofen and celecoxib), and acetaminophen (Montilla-García et al., 2017). Interestingly, with the exception of oxycodone, which showed a similar potency in reversing tactile allodynia and grip strength deficits, the dose of all other known analgesic drugs was lower for functional deficits than for cutaneous hypersensitivity (Montilla-García et al., 2017). Here we show that morphine was less potent in reversing grip strength deficits than tactile allodynia, which supports the notion that the pharmacological sensitivity of these two outcomes during inflammation is not identical. Differences between the analgesic sensitivity of standard cutaneous measures of pain and pain-induced functional deficits have been described previously for other outcomes used to test pain interference on physical function, such as inflammation-induced weight bearing differences or wheel running depression (reviewed in Cobos and Portillo-Salido, 2013).

We also tested the effects of S1RA (in the absence of morphine) on nociceptive heat pain, inflammatory tactile allodynia and grip strength deficits induced by inflammation. S1RA did not alter nociceptive heat pain, as previously reported for this and other sigma-1 antagonists (e.g., Chien and Pasternak, 1994, 1995; Tejada et al., 2014, 2017). Here we show that this sigma-1 antagonist was able to markedly ameliorate sensory hypersensitivity during mild inflammation, as previously reported (Parenti et al., 2014; Gris et al., 2015; Tejada et al., 2018), but it was devoid of effect when inflammation was more prominent (at a higher dose of CFA). We previously reported a clear relationship between ankle thickness and the volume of CFA administered (Montilla-García et al., 2017). We needed to use a higher dose of CFA in our study because the decreases in grip strength induced by lower doses of this compound were too small to reliably assess the effects of analgesic drugs, as we previously reported (Montilla-García et al., 2017). Therefore, the experimental conditions used here to induce inflammatory allodynia were too restrictive to detect the antiallodynic effects induced by sigma-1 antagonism. Despite the absence of effect of S1RA on inflammatory tactile allodynia during this more prominent inflammation, here we show that S1RA was able to partially ameliorate grip strength deficits induced by inflammation, which again indicates that the sensitivity to drug effects differs between tactile allodynia and grip strength deficits. We have previously shown that both nociceptive heat pain and tactile allodynia during inflammation in our experimental conditions are sensitive to the *in vivo* ablation of transient receptor potential vanilloid (TRPV1)-expressing neurons by resiniferatoxin (Montilla-García et al., 2017, 2018). However, grip strength deficits during inflammation are insensitive to resiniferatoxin treatment (Montilla-García et al., 2017). These results indicate that the neurobiological mechanisms involved in grip strength deficits during inflammation and in the behavioral tests of cutaneous sensitivity explored here are different. Therefore, the effect of S1RA on grip strength deficits in mice with inflammation may be due to sigma-1 actions in other pain pathways unrelated to those involved in heat nociceptive pain or inflammatory tactile allodynia.

In this study we show that the systemic administration of S1RA was able to enhance morphine antinociception to contact heat stimulation, in agreement with previous reports which used other types of nociceptive heat stimulus (reviewed in Sánchez-Fernández et al., 2017). We show that S1RA markedly potentiated the antiallodynic effect of morphine in mice with inflammation. To our knowledge this is the first reported evidence that sigma-1 antagonism enhances the effect of an opioid drug in a pathological pain model. The enhancement by S1RA of the effects of morphine on nociceptive heat pain and inflammatory tactile allodynia was abolished by the administration of the sigma-1 agonist PRE-084, which argues in favor of an action mediated by sigma-1 receptors in these S1RA-induced effects. Despite the evident increase in the effects of morphine on nociceptive heat pain and inflammatory tactile allodynia noted above, and despite the greater sensitivity of grip strength deficits to the effects of S1RA when administered alone (in the absence of morphine), we show that S1RA was not able to potentiate the effects of morphine on grip strength deficits induced by inflammation. This result further supports the notion that different neurobiological mechanisms are involved in grip strength deficits and cutaneous pain.

We also explored the modulation of morphine analgesic tolerance by S1RA, given that this unavoidable opioid effect is a substantial drawback to the use of opioid analgesics (Morgan and Christie, 2011). We found that when S1RA was administered to morphine-tolerant mice, it was able to rescue the effect of morphine on nociceptive pain in response to contact heat stimulus, in agreement with previous studies that used nociceptive heat stimulation (Vidal-Torres et al., 2013; Rodríguez-Muñoz et al., 2015). We also show that S1RA was able to rescue the effects of morphine on inflammatory tactile allodynia. The rescue of morphine tolerance by this sigma-1 antagonist in heat nociception or inflammatory tactile allodynia was abolished by the administration of PRE-084, which again argues in favor of a role for an action mediated by sigma-1 receptors in the effects induced by S1RA. The association of S1RA and morphine administered on the day of the behavioral evaluation to morphine-tolerant animals also induced a clear recovery of grip strength deficits. It is worth pointing out the possibility that not all the effect detected in morphine-tolerant animals that received S1RA + morphine on the day of the experiment were due to the rescue of morphine tolerance; given that S1RA had a slight but significant effect on grip strength in mice with inflammation, this might have contributed to the effect observed. We show that this effect of S1RA + morphine in tolerant animals was reversed not only by the administration of PRE-084, but also by the administration of (+)-pentazocine, another selective sigma-1 agonist (Cobos et al., 2008), which further supports the selectivity of the effects induced by S1RA.

These results on the rescue of morphine tolerance by S1RA cannot be explained by pharmacokinetic interactions that might increase the level of morphine or its major murine metabolite morphine-3-glucuronide (Pasternak and Pan, 2013), since we found that the levels of these compounds remained unaltered after acute S1RA administration in tolerant animals. Our results point instead to pharmacodynamic interactions between sigma-1 antagonism and opioid effects. Interestingly, plasma levels of S1RA in our mice were similar to plasma levels of this drug found in humans after oral S1RA treatment at therapeutic doses (Abadias et al., 2013; Bruna et al., 2018). To study whether the administration of S1RA was able to prevent the development of morphine tolerance, we administered this sigma-1 antagonist during an escalating morphine dosage regimen (but not on the day of the behavioral evaluation). We found that, as previously reported, the repeated, pre-emptive administration S1RA failed to prevent tolerance to the effects of morphine on a nociceptive heat stimulus (Vidal-Torres et al., 2013). In addition, sigma-1 antagonism also failed to prevent tolerance to the antiallodynic effect of morphine during inflammation. The repeated administration of S1RA did not alter grip strength in morphine-tolerant animals with inflammation, although surprisingly, it completely prevented the development of tolerance to the effect of morphine on grip strength deficits. Therefore, the effects induced by S1RA in the preemptive protocol cannot be attributed to the effects of this compound alone. Interestingly, we found that at the time of the behavioral tests, the levels of morphine or morphine-3-glucuronide remained unaltered in S1RA-treated mice and there was no remaining S1RA in either plasma or brain tissues. These results indicate that S1RA, when repeatedly administered with morphine, is able to induce protective effects against the development of tolerance.

It has been suggested that sigma-1 antagonism both potentiates opioid analgesia and diminish morphine tolerance by decreasing the inhibition of μ-opioid receptors by *N*-methyl-d-aspartate receptor (NMDAR) activity (Rodríguez-Muñoz et al., 2015). Because S1RA failed to enhance the effect of morphine on grip strength deficits during inflammation in non-tolerant mice but was able to successfully prevent morphine tolerance in this outcome, the mechanisms for opioid potentiation by sigma-1 receptors appear to be dissociated from the effects on opioid tolerance, at least in this measure of pain-induced functional impairment.

It has been reported that the sigma-1 antagonists S1RA and BD-1063 prevent paclitaxel-induced neuropathic pain (Nieto et al., 2012, 2014), which points to broader neuroprotective effects of sigma-1 antagonism. However, it remains unclear why this prevention of morphine tolerance by S1RA affects only grip strength deficits but not heat nociception or inflammatory tactile allodynia. Although the mechanisms of cutaneous sensitivity have been extensively explored for decades, little is known about the mechanisms of pain-induced functional impairment, which may not fully overlap (Cobos and Portillo-Salido, 2013; Montilla-García et al., 2017; Negus, 2018). Similarly, the mechanisms of opioid analgesic tolerance might also depend on the pain measure used. Regardless of the exact mechanisms involved in the differential results obtained in grip strength deficits and the other two pain measures explored in the present study, in light of our results it is clear that they are not fully equivalent.

Interestingly, neither the acute administration of morphine nor the acute or repeated administration of S1RA were able to alter CFA-induced inflammatory edema. The results with S1RA are in agreement with a previous study showing that this sigma-1 antagonist did not alter carrageenan-induced inflammatory edema (Gris et al., 2014; Tejada et al., 2014). Therefore, taking into account the amelioration of grip strength deficits by the drugs tested here but their lack of effects on inflammatory edema, our results suggest that their effects on grip strength are not attributable to improved grip strength resulting from reduced swelling around the joints or tendons, and that swelling *per se* does not prevent movement. Furthermore, grip strength is classically used to assess neurotoxi in rodents, and is included in the Irwin screen (Irwin, 1968; Mattsson et al., 1996), which is ingrained in the pharmaceutical industry as the first tier of preclinical testing to detect drug-induced neurotoxic effects (Moser, 2011). We show that morphine or S1RA (administered acutely or repeatedly), as well as the acute administration of PRE-084, did not alter grip strength in animals without inflammation, suggesting that the doses used in our study did not alter normal motor function. Taken together, our results suggest that the drugs tested here exert their effects through pain modulation rather than through unspecific effects on inflammation or motor performance.

Although von Frey testing, the behavioral assay currently used most widely in preclinical pain research, is undoubtedly useful to detect sensory alterations in patients with neuropathy (e.g., Bennett, 2001; Bouhassira et al., 2005; Moharic et al., 2012), it is almost never used in other human pain conditions such as rheumatic disease. Therefore, although von Frey testing has been established as the standard in preclinical pain testing, it is not a widely used pain measure in patients with non-neuropathic chronic painful diseases. On the other hand, grip strength has been widely and routinely evaluated for decades as a functional measure in patients with joint inflammation caused by rheumatic disease (e.g., Bijlsma et al., 1987; Pincus and Callahan, 1992; Lee, 2013), and it is known to correlate with pain (Callahan et al., 1987; Fraser et al., 1999; Overend. T. J et al., 1999). In fact, one set of consensus-based recommendations advocates measuring physical function as one of the main outcomes in clinical trials of pain treatments (Dworkin et al., 2008). In light of the differences we observed in the effects of sigma-1 receptors on opioid analgesia and tolerance, as reflected in grip strength tests and the measures of cutaneous sensitivity explored in the present study, we believe measures of physical functioning merit inclusion in the standard repertoire of behavioral tests in preclinical laboratories, to better approximate the human pain phenotype in preclinical pain research.

We conclude that sigma-1 receptors play a pivotal role in the control of morphine analgesia and tolerance, albeit in a manner dependent on the type of painful stimulus explored. These findings may have important therapeutic implications for the use of sigma-1 antagonists as opioid adjuvants. In addition, the results we obtained for grip strength deficit as a surrogate pain measure were not equivalent to those seen when standard measures of cutaneous sensitivity were used. Further studies are needed to fully understand the mechanisms through which pain interferes with physical function.

## Author Contributions

EC designed the research. ÁM-G, MT, MR-C, SY, and IB-C performed the research. ÁM-G, MT, SY, and DZ analyzed the data. ÁM-G, MT, MR-C, SY, IB-C, DZ, and EC, wrote the manuscript. All authors read and approved the final version of the manuscript.

## Conflict of Interest Statement

The authors declare that the research was conducted in the absence of any commercial or financial relationships that could be construed as a potential conflict of interest.
