# Supplementary material for: Modulation by Sigma-1 Receptor of Morphine Analgesia and Tolerance: Nociceptive Pain, Tactile Allodynia and Grip Strength Deficits During Joint Inflammation
Source: Front Pharmacol. 2019 Feb 22;10:136. doi: 10.3389/fphar.2019.00136 (PMC6395397; doi:10.3389/fphar.2019.00136)
Supplement: Supplementary file 1 [file Data_Sheet_1.PDF]

Supplementary Material

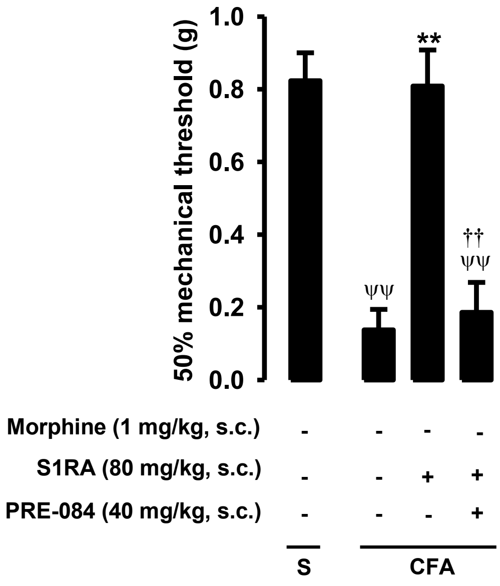

Suppl. Fig. 1. Effects of S1RA alone or associated with PRE-084 on mechanical allodynia induced by mild inflammation. The results represent the 50% mechanical threshold (determined with von Frey filaments) in mice treated periarticularly (10 μL/paw) with CFA or saline (S). S1RA (80 mg/kg) or its vehicle was subcutaneously (s.c.) administered alone or associated with PRE-084 (40 mg/kg, s.c.) or its vehicle. Each bar and vertical line represent the mean ± SEM of values obtained in 8–10 animals. Statistically significant differences between the values obtained in: animals with and without inflammation ($^{\psi\psi}P < 0.01$); animals treated with S1RA or its vehicle (**P < 0.01); S1RA associated with PRE-084 or its vehicle ($^{\dagger\dagger}P < 0.01$) (one-way ANOVA followed by Student–Newman–Keuls test).

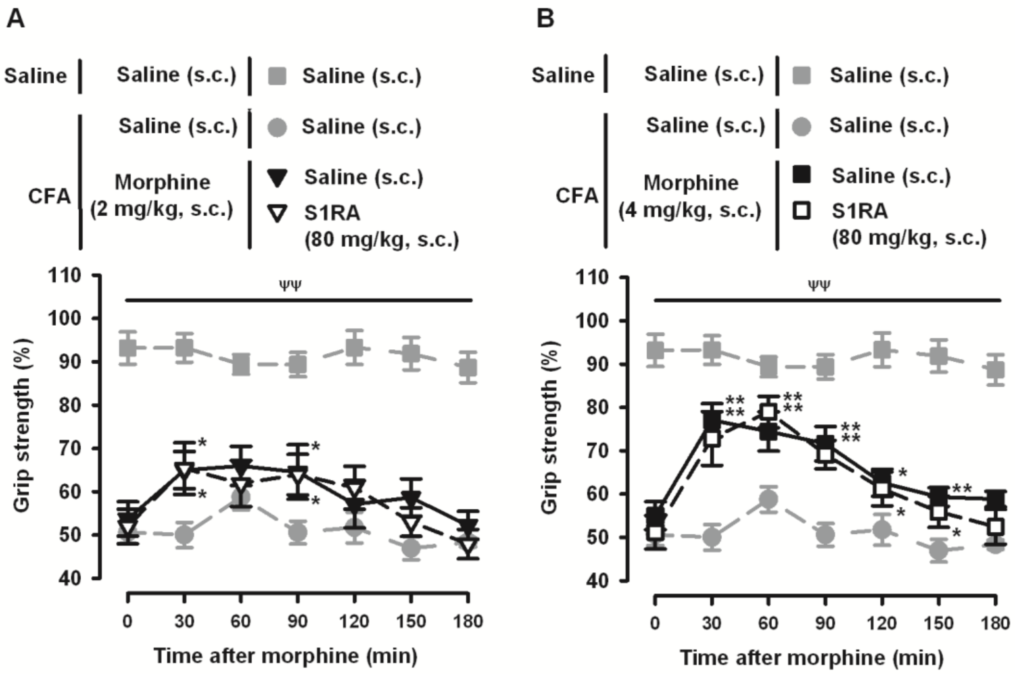

Suppl. Fig. 2. Time-course of the effects of morphine and its association with S1RA on grip strength deficits induced by inflammation. The results represent grip strength values (expressed as the percentage of the basal value in each individual mouse before the injection) treated periarticularly with CFA or saline. (A) Time-course of the effects of the subcutaneous (s.c.) administration of morphine 2 mg/kg associated with S1RA (80 mg/kg) or its vehicle. (B) Time-course of the effects of the s.c. administration of morphine 4 mg/kg associated with S1RA (80 mg/kg) or its vehicle. (A and B) Each point and vertical line represent the mean ± SEM of values obtained in 8–10 animals. Statistically significant differences between the values in mice with inflammation treated with the drug or vehicle at each time-point: *$P < 0.05$, **$P < 0.01$. There were no statistically significant differences between the values in mice treated with morphine associated with S1RA or its vehicle at any time-point tested (two-way repeated measures ANOVA followed by Student–Newman–Keuls test).
